# Effects of Epigallocatechin Gallate (EGCG) on Urinary Bladder Urothelial Carcinoma―Next-Generation Sequencing and Bioinformatics Approaches

**DOI:** 10.3390/medicina55120768

**Published:** 2019-12-01

**Authors:** Hsiang-Ying Lee, Yi-Jen Chen, Wei-An Chang, Wei-Ming Li, Hung-Lung Ke, Wen-Jeng Wu, Po-Lin Kuo

**Affiliations:** 1 Graduate Institute of Clinical Medicine, College of Medicine, Kaohsiung Medical University, Kaohsiung 807, Taiwan; ashum1009@hotmail.com (H.-Y.L.); chernkmu@gmail.com (Y.-J.C.); 960215kmuh@gmail.com (W.-A.C.); 2 Department of Urology, Kaohsiung Municipal Ta-Tung Hospital, Kaohsiung 801, Taiwan; wejewu@kmu.edu.tw; 3 Department of Urology, Kaohsiung Medical University Hospital, Kaohsiung 807, Taiwan; u8401067@yahoo.com.tw (W.-M.L.); hunglungke@gmail.com (H.-L.K.); 4 School of Medicine, College of Medicine, Kaohsiung Medical University, Kaohsiung 807, Taiwan; 5 Department of Physical Medicine and Rehabilitation, Kaohsiung Medical University Hospital, Kaohsiung 807, Taiwan; 6 Division of Pulmonary and Critical Care Medicine, Kaohsiung Medical University Hospital, Kaohsiung 807, Taiwan; 7 Department of Urology, Ministry of Health and Welfare Pingtung Hospital, Pingtung 900, Taiwan; 8 Center for Cancer Research, Kaohsiung Medical University, Kaohsiung 807, Taiwan

**Keywords:** bladder cancer, epigallocatechin gallate, next-generation sequencing, bioinformatics, mRNA, microRNA

## Abstract

*Background and objectives:* Bladder urothelial carcinoma is the most common type of genitourinary cancer. Patients with bladder cancer may have limited treatment efficacy related to drug toxicity, resistance or adverse effects, and novel therapeutic strategies to enhance treatment efficacy or increase sensitivity to drugs are of high clinical importance. Epigallocatechin gallate (EGCG) is a polyphenolic compound found in green tea leaves, and a potential anti-cancer agent in various cancer types through modulating and regulating multiple signaling pathways. The current study aimed to explore the role and novel therapeutic targets of EGCG on bladder urothelial carcinoma. *Materials and Methods:* The BFTC-905 cells, human urinary bladder transitional cell carcinoma (TCC) cell line, were treated with EGCG or water for 24 hours, and the expression profiles of mRNAs and microRNAs were analyzed using next generation sequencing (NGS). The enriched biological functions were determined using different bioinformatics databases. *Results:* A total of 108 differentially expressed genes in EGCG-treated bladder TCC cells were identified, which were mainly involved in nicotinamide adenine dinucleotide (NAD) biogenesis, inflammatory response and oxidation-reduction metabolism. Moreover, several microRNA-mRNA interactions that potentially participated in the response of bladder TCC to EGCG treatment, including miR-185-3p- *ARRB1* (arrestin beta 1), miR-3116- *MGAT5B* (alpha-1,6-mannosylglycoprotein 6-beta-N-acetylglucosaminyltransferase B), miR-31-5p-*TNS1* (tensin 1), miR-642a-5p-*TNS1*, miR-1226-3p- *DLG2* (discs large homolog 2), miR-484-*DLG2*, and miR-22-3p- *PPM1K* (protein phosphatase 1K). *Conclusions:* The current findings provide insights into novel therapeutic targets and underlying mechanisms of action of EGCG treatment in bladder cancer.

## 1. Introduction

Transitional cell carcinoma (TCC), also called urothelial carcinoma, is the most common type of genitourinary cancer. Common risk factors include cigarette smoking and chemical exposure. Worldwide, the annual incidence rates are approximately 9.0 and 2.2 per 100,000 persons for men and women, respectively; the annual mortality rates are reported to be 3.2 and 0.9 per 100,000 persons for men and women, respectively [1]. However, the incidence and mortality rates of bladder cancer vary across countries. For instance, in Taiwan, higher incidence of bladder cancer was found in black-foot disease endemic areas [2]. The recommendations on the clinical management of bladder cancer is based on its muscle invasiveness, classified as non-muscle-invasive or muscle-invasive bladder cancer. Approximately 75% of patients with newly diagnosed bladder cancer present with non-muscle-invasive disease and undergo transurethral resection of the bladder followed by adjuvant therapy, such as intravesical chemotherapy or bacillus Calmette–Guérin (BCG) immunotherapy, to reduce the risk of disease recurrence or progression [1]. In patients with muscle-invasive disease or disease progression, treatment options include radical cystectomy and neoadjuvant chemotherapy, but as patients potentially progressed to metastatic status, the survival rate drops [3]. The efficacy of chemotherapy may be limited by drug toxicity, resistance, and adverse effects, and so novel therapeutic strategies that enhance treatment efficacy or increase sensitivity to drugs are of high clinical importance.

Epigallocatechin gallate (EGCG) is a major bioactive polyphenolic compound found in green tea leaves, and the effect of EGCG and their synthetic analogs have been reported as potential anti-cancer agents in various cancer types through modulating and regulating multiple signaling pathways [4]. Regarding the potential effects of EGCG on the urinary tract system, flavonoid antioxidants including EGCG were reported to ameliorate cyclophosphamide-induced cystitis [5]. In addition, Bazi et al. claimed the protective effect of EGCG against bladder degeneration in rats [6], and the antioxidant effect against H_2_O_2_-induced oxidative stress through superoxide was observed in normal and malignant bladder cells [7]. In bladder cancer, EGCG inhibits proliferation and migration, and induces apoptosis of bladder cancer cells [8,9], and it also exerts cytotoxic effect and prevents intravesical tumor growth with similar efficacy to mitomycin C [10]. Sterilization through radiation could keep EGCG in a stable form and of benefit in treating superficial bladder cancer [11]. A recent phase II trial of polyphenol consisted primarily of EGCG administered in patients prior to bladder cancer surgery resulted in definite tissue accumulation and biologic activity of polyphenol [12]. The evidence suggested EGCG as a potential therapeutic agent for prevention of tumor implantation in bladder cancer.

To investigate the role and novel therapeutic targets of EGCG on bladder urothelial carcinoma, we conducted this study to explore the effect of EGCG on altered gene expression and microRNA expression profiles of bladder cancer cells using next generation sequencing (NGS), and further investigate functionally enriched biological themes using different bioinformatics database analyses.

## 2. Materials and Methods

### 2.1. Cell Culture and RNA Extraction

The BFTC-905 cells, human urinary bladder transitional cell carcinoma (TCC) cell line, purchased from Leibniz Institute DSMZ (German Collection of Microorganisms and Cell Cultures GmbH, Braunschweig, Germany), were cultured in 6 cm culture plates with Dulbecco’s Modified Eagle Medium and incubated in 37 °C incubator containing 5% CO_2_. After 24 h of incubation, cells were treated either with vehicle alone or 100 μM of EGCG for 24 h. The cells were then harvested and total RNA extracted using TRIzol Reagent (Thermo Fisher Scientific, Waltham, MA, USA), following instruction from the manufacturer. The quantity of extracted RNA was analyzed using ND-1000 spectrophotometer (NanoDrop Technologies, Wilmington, DE, USA) and the quality of RNA was analyzed using Bioanalyzer 2100 (Agilent Technologies, Santa Clara, CA, USA).

### 2.2. Next-Generation Sequencing

For NGS, library preparation and deep sequencing were carried out at Welgene Biotechnology Company (Taipei, Taiwan) following the protocol of Illumina (San Diego, CA, USA). In brief, sequencing for transcriptome was determined using sequencing-by-synthesis technology, and the sequencing data was generated by Welgene’s pipeline based on Illumina’s base calling program bcl2fastq v2.2.0. Trimmomatic version 0.36 was used for adaptor clipping and sequence quality trimming [13]. The read alignment was accomplished using HISAT2 to map sequencing reads to genomes [14], followed by differential expression analysis using Cuffdiff (Cufflinks version 2.2.1) [15] with genome bias detection/correction and Welgene in-house programs. The genes with low expression levels (<0.3 fragment per kilobase of transcript per million mapped reads (FPKM)) in both EGCG-treated and control BFTC-905 cells were excluded. The *p*-values were calculated by Cuffdiff with non-grouped sample using “blind mode”, where all samples were determined as replicates of a single global “condition” to build one model for statistical test [15]. Genes with *p*-values < 0.05 and fold-change >2.0 were determined as significantly differentially expressed. For small RNA sequencing, samples were prepared using Illumina sample preparation kit, following the TruSeq Small RNA Sample Preparation Guide. Reverse transcription and PCR amplification was performed after 3′ and 5′ adaptor ligation to RNA. The amplified cDNA constructs were size-fractionated and purified using 6% polyacrylamide gel electrophoresis. The bands containing 18–40 nucleotide RNA fragments were extracted and sequenced on an Illumina instrument with 75 base pair single-end reads. Sequencing data was processed with the Illumina software and trimmed for qualified reads using Trimmomatic version 0.36 [13]. The qualified reads were analyzed using miRDeep2 to clip the 3′ adapter sequence and eliminate reads shorter than 18 nucleotides [16]. The reads were then aligned to the human genome from the University of California, Santa Cruz (UCSC). Only reads that mapped perfectly to the genome five or less times were used for miRNA detection. The miRNAs with low expression levels (<1 normalized read per million (RPM)) in both EGCG-treated and control BFTC-905 cells were excluded. Those miRNAs with fold-change >2.0 were determined as differentially expressed.

### 2.3. Bioinformatics Databases

To determine the enriched biological functions and interaction networks of the differentially expressed genes, several bioinformatics databases were used, including Ingenuity Pathway Analysis (IPA) [17], OmicsNet [18], and Database for Annotation, Visualization and Integrated Discovery (DAVID) [19]. In addition, to predict miRNA targets, miRmap target prediction database (version 1.1) [20] was used, and those candidate miRNA–mRNA interactions were validated in two other target prediction databases, TargetScan [21] and miRDB [22].

## 3. Results

This section may be divided by subheadings. It should provide a concise and precise description of the experimental results, their interpretation as well as the experimental conclusions that can be drawn.

### 3.1. Identification of Differentially Expressed Genes in EGCG-Treated Bladder TCC Cells

**Figure 1:**
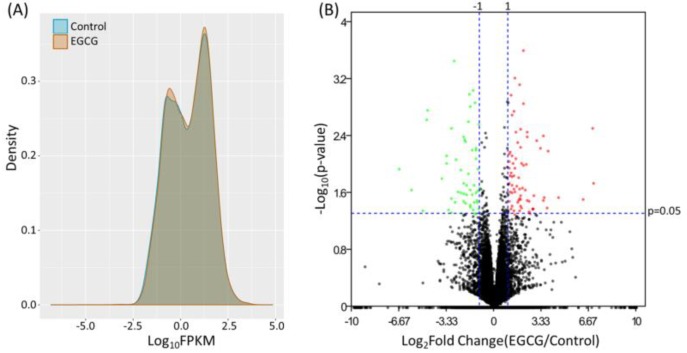
Differential gene expression patterns of BFTC-905 cells, human urinary bladder transitional cell carcinoma (TCC) cell line, treated with epigallocatechin gallate (EGCG) or water (control). (**A**) The density plot showing the frequency distribution of fragments per kilobase of transcript per million mapped reads (FPKM) in bladder TCC cells treated with EGCG or water (control). (**B**) The volcano plot showing the differentially expressed genes in bladder TCC cells treated with EGCG or water (control). Genes significantly up-regulated in EGCG-treated bladder TCC cells were plotted in red, and genes significantly down-regulated were plotted in green. Genes with fold-changes >2.0 and *p*-value < 0.05 were considered significantly dysregulated.

**Figure 2:**
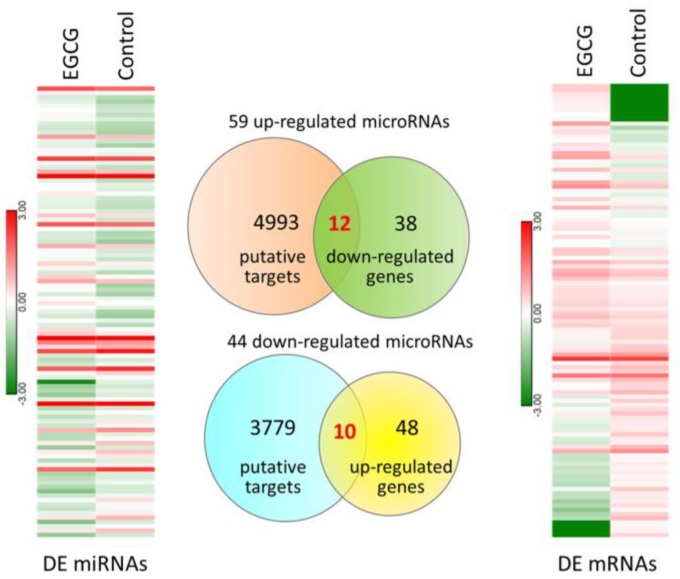
The heatmaps of differentially expressed (DE) miRNAs and DE mRNAs in EGCG-treated bladder TCC cells were shown in left and right panels, respectively. The putative targets of dysregulated miRNAs in EGCG-treated bladder TCC cells were predicted from the miRmap database, and those with miRmap scores ≥97.0 were selected. The putative targets were then matched to significantly differentially expressed mRNAs in EGCG-treated bladder TCC cells, and the Venn diagram was shown in the middle panel. A total of 22 candidate genes with potential miRNA interactions were identified.

The expression profile of EGCG-treated and control bladder TCC cells were obtained from NGS results, and the FPKM performance between the two samples were presented in density plot in Figure 1A. To identify differentially expressed genes in EGCG-treated bladder TCC cells, the dysregulated genes were displayed in volcano plot (Figure 1B), and significantly dysregulated genes were selected under the criteria of at least two-fold change in EGCG treated cells and significant differential expression with *p* < 0.05. A total of 58 up-regulated and 50 down-regulated genes in EGCG-treated bladder TCC cells were identified. The heatmap of these 108 dysregulated genes were shown in the right panel of Figure 2.

### 3.2. Exploring Potentially Altered miRNA–mRNA Interactions in EGCG-Treated Bladder TCC Cells

The miRNA expression profile of EGCG-treated and control bladder TCC cells were simultaneously performed using NGS. Dysregulated miRNAs were selected under the criteria of at least two-fold change in EGCG treated cells and normalized read counts of more than 1 RPM. There were 59 up-regulated and 44 down-regulated miRNAs identified in EGCG-treated bladder TCC cells, and the heatmap of these 103 dysregulated miRNAs were displayed in the left panel of Figure 2. The putative targets of these dysregulated miRNAs were obtained from miRmap database, selecting those with miRmap scores ≥97.0 indicating high repression strength. The putative targets were then matched to significantly differentially expressed genes identified in the previous section. The intersection Venn diagram was shown in the middle panel of Figure 2, where 22 candidate genes with potential miRNA interactions were identified. A list of the 22 candidate genes along with their expression levels were shown in Table 1.

### 3.3. The 22 Candidate Genes were Involved in NAD Biosynthesis, Inflammatory Response and Cancer

To understand the involvement of the 22 candidate genes with potential miRNA interactions in EGCG-treated bladder TCC cells, these genes were uploaded to IPA software for core analysis. The top five canonical pathways were mainly related to nicotinamide adenine dinucleotide (NAD) biosynthesis; additionally, most of these genes were related to diseases and disorders in inflammatory response and cancer (Table 2). The network analysis grouped these genes into three sub-networks, where 20 genes were related to nervous system function and inflammatory response, as listed in Table 3. The activation z-score was −0.128 for inflammatory response, which indicated inhibited inflammatory response among these candidate genes.

**Figure 3:**
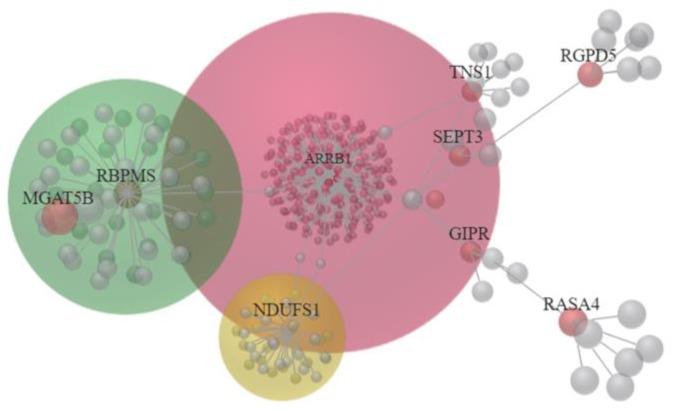
The protein–protein interaction (PPI) network of 22 candidate genes with potential miRNA interactions was generated from OmicsNet using InnateDB database. A module-based explorer using WalkTrap algorithm indicated major subnetworks among the PPI network. *ARRB1*, *MGAT5B*, *RBPMS*, and *NDUFS1* were identified as central hubs of these major subnetworks.

**Figure 4:**
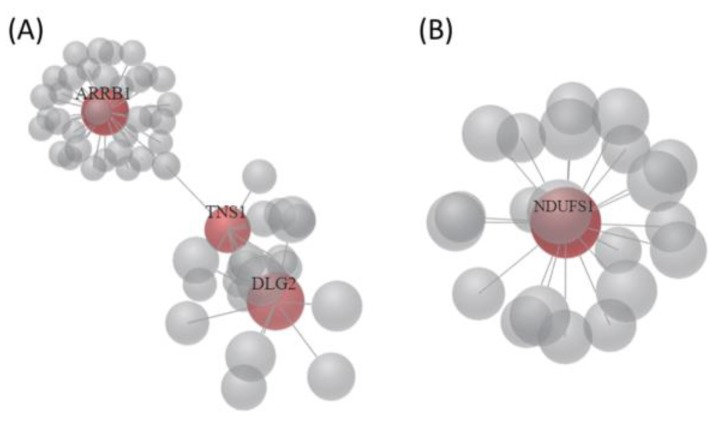
Two major PPI networks of 22 candidate genes with potential miRNA interactions were generated from OmicsNet using the STRING database. (**A**) *ARRB1*, *TNS1*, and *DLG2* were central hub genes of major PPI network 1; (**B**) *NDUFS1* was the central hub gene of major PPI network 2.

**Figure 5:**
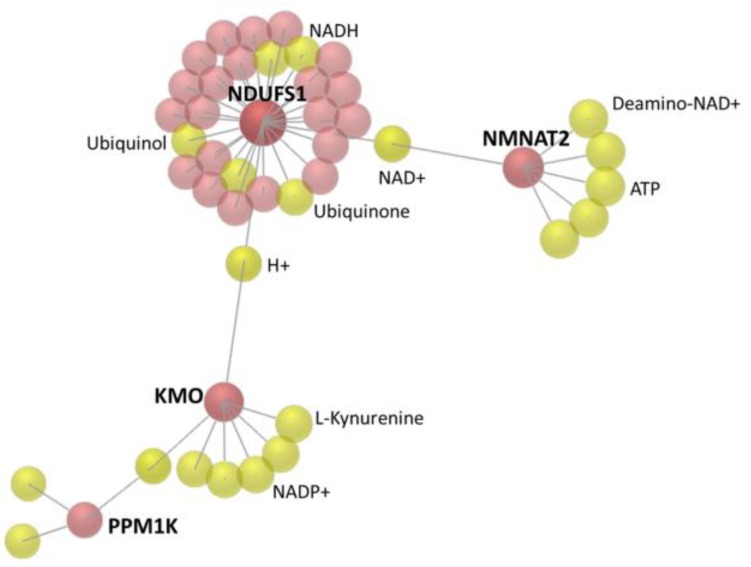
The metabolite–protein interaction data was generated from OmicsNet, considering all Kyoto Encyclopedia of Genes and Genomes (KEGG) database reactions. *NDUFS1*, *KMO*, and *NMNAT2* showed close interactions with nicotinamide adenine dinucleotide (NAD) metabolites.

To investigate the interaction network among these candidate genes, the OmicsNet was used for protein–protein interaction (PPI) network generation using both InnateDB and STRING databases. The major PPI subnetwork from InnateDB was displayed in Figure 3, in which module-based explorer using WalkTrap algorithm identified *ARRB1*, *MGAT5B*, *RBPMS*, and *NDUFS1* as central hubs of major subnetworks. Similarly, *ARRB1* and *NDUFS1* were also identified as central hubs among the two major PPI subnetworks from the STRING database (Figure 4). In addition, metabolite-protein interaction data was also investigated on all KEGG reactions in the OmicsNet. Among the interaction network, *NDUFS1*, *KMO*, and *NMNAT2* were found closely associated with NAD related metabolites, as shown in Figure 5.

### 3.4. Enriched Biological Functions Among Differentially Expressed Genes in EGCG-Treated Bladder TCC Cells

To investigate functionally enriched biological themes among the 108 differentially expressed genes in EGCG-treated bladder TCC cells, IPA and DAVID databases were used. The IPA result identified NAD biosynthesis (*p* = 1.77 × 10^−3^) and Hippo signaling (*p* = 6.74 × 10^−3^) being the top canonical pathways, and cancer the top disease involved in these dysregulated genes (Table 4). In the DAVID database analysis, the significantly enriched biological processes were also observed to be associated with oxidation-reduction process and NFκB signaling, and borderline significance in NAD metabolic process and metabolic pathways (Table 5).

### 3.5. Identification of Potential miRNA Interactions in EGCG-Treated Bladder TCC Cells

To identify miRNAs potentially regulating the differentially expressed genes in EGCG-treated bladder TCC cells, several miRNA target prediction databases were used to determine miRNAs potentially regulating major hubs of the previously identified interaction networks, including *ARRB1*, *RBPMS*, *MGAT5B*, *NDUFS1*, *TNS1*, *DLG2*, *NMNAT2*, *KMO*, and *PPM1K*. Matching to the differentially expressed miRNAs in EGCG-treated bladder TCC cells, several of the miRNA regulations were consistently validated in miRmap, TargetScan and miRDB databases. The potential miRNA interactions were listed in Table 6.

## 4. Discussion

**Figure 6:**
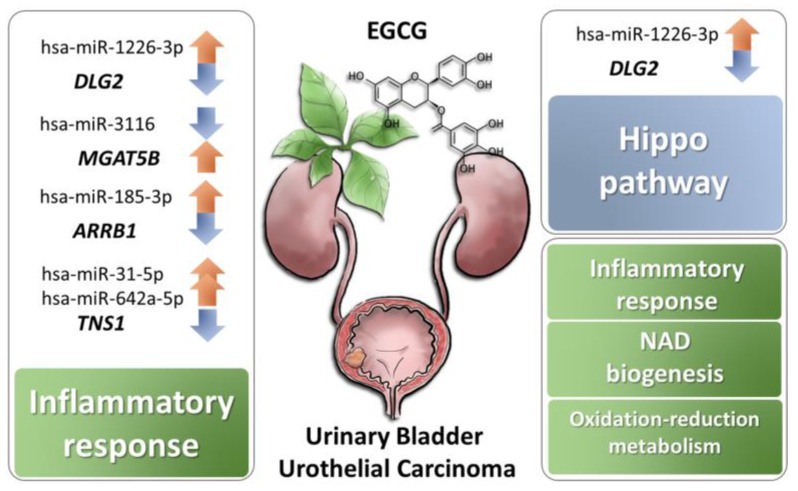
Schematic summary of potential effects of EGCG on bladder urothelial carcinoma.

The current study investigated the potential effects of EGCG on bladder urothelial carcinoma using NGS and bioinformatics analysis. A total of 108 differentially expressed genes in EGCG-treated bladder TCC cells were identified, and were mainly involved in NAD biogenesis, inflammatory response, and oxidation-reduction metabolism. In addition, miRNA target prediction analysis identified several miRNA–mRNA interactions that potentially participated in the response of bladder TCC to EGCG treatment, including miR-185-3p-*ARRB1*, miR-3116-*MGAT5B*, miR-31-5p-*TNS1*, miR-642a-5p-*TNS1*, miR-1226-3p-*DLG2*, miR-484-*DLG2*, and miR-22-3p-*PPM1K*. Of these genes with potential miRNA interactions, *DLG2* was involved in Hippo pathway and inflammatory response, and *TNS1*, *ARRB1*, and *MGAT5B* were involved in inflammatory response. A schematic summary of the current study is illustrated in Figure 6.

NAD is an essential coenzyme participating in various energy metabolic pathways, cell signaling regulatory pathways and DNA repair, and a potential therapeutic target for oncotherapy [23]. Higher levels of NAD in cancer cells has been reported [24]. The high glycolytic rate of cancer cells, called “Warburg effect” requires NAD for glycolytic enzymes, and the NAD salvage pathway is important in cancer biology and an intriguing target to prevent cancer growth [23]. Nicotinamide nucleotide adenylyltransferase 2 (NMNAT2) is a cytosolic enzyme that assists in maintaining mitochondrial NAD level, although the association between NAD salvage pathway and mitochondrial NAD level remains unclear [23]. In osteosarcoma cells, knockdown of NMNAT2 reduced p53-mediated cell death upon DNA damage [25]. Interestingly, previous study has reported overexpression of NMNAT2 enhanced sensitivity of colorectal cancer cells to specific pro-drug and improved therapeutic efficacy [26]. In addition, reduced NAD level also activates signaling pathways promoting epithelial to mesenchymal transition (EMT), facilitating tumor progression [27]. In bladder cancer, the role of nicotinamide metabolism has recently been reported. The concentration of NAD in whole blood was decreased in patients with bladder cancer compared to control subjects, and plasma levels of nicotinamide metabolites were also altered [28]. NQO1, one of the major quinone reductases highly inducible under cellular stress, modulate NAD/NADH redox balance, and specific polymorphism of NQO1 is associated with cancer risk in urinary system [29]. However, the role of NMNAT2 in bladder cancer cells and whether expression of NMNAT2 affects therapeutic efficacy remains unclear.

Another important issue is reactive oxygen species (ROS) which is toxic to all cells, including cancer cells. Therefore, a good anti-oxidant defense system in cells is essential to prevent cytotoxic damage [23]. NAD is also involved in the regulation of oxidative stress, and report showed ascorbate induced cell death of neuroblastoma cells through NAD depletion and ROS-induced DNA damage, which suggested that NAD and ROS production are partially responsible for the cancer cell cytotoxicity [30]. The current evidence suggested the importance of NAD biosynthesis in cancer pathogenesis and NAD as potential biomarkers. Our analytic results also indicated the involvement of NAD biosynthesis and related oxidation-reduction process in EGCG-treated bladder TCC cells, which suggested the potential effect of EGCG in urothelial carcinoma through alteration in NAD and oxidation-reduction related metabolism.

Chronic inflammation plays an important role in cancer. The link between inflammation and development of bladder cancer has also been reported, and the oncogenic changes may potentiate inflammatory microenvironment that leads to angiogenesis and invasion of bladder cancer [31,32]. EGCG is a major constituent of green tea polyphenols that is documented as having antioxidant, anti-inflammatory, and anti-cancer effects [33]. The anti-inflammatory property is mainly through targeting toll-like receptor 4 signaling pathway, which is also associated with cancer progression [34]. In our current study, results suggested the involvement of inflammatory response among dysregulated genes with potential miRNA interactions, particularly *TNS1*, *MGAT5B*, and *ARRB1*, and the activation z-score was −0.128, indicating inhibited inflammatory response upon EGCG treatment.

Arrestin beta 1 (ARRB1) is one of the beta arrestins regulating various cellular physiologic processes, and have been demonstrated to have pro-tumorigenic effects [35,36]. Kallifatidis et al. recently reported increased ARRB1 expression in bladder cancer cells, and ARRB1 gene knockout reversed the aggressive phenotype of bladder cancer cell lines [37]. MiR-185-3p is a potential oncosuppressor miRNA evident in nasopharyngeal carcinoma (NPC), and the inhibition of miR-185-3p promoted invasion and metastasis of NPC cells [38]. In the current study, altered miR-185-3p-ARRB1 interaction was identified in EGCG-treated bladder TCC cells, with 3.92-fold up-regulated miR-185-3p and 4.01-fold suppressed ARRB1. The evidence suggested the potential effect of EGCG in altered miR-185-3p expression that may affect bladder TCC cells.

Down-regulation of tensin 1 (TNS1) potentially regulated by miR-31-5p and miR-642a-5p was also identified in the current study. The expression of TNS1 was found higher in colorectal cancer cell lines and tissues, and suppression of TNS1 decreased proliferation and invasiveness of cancer cells, while the level of TNS1 was positively associated with poor survival in patients with colorectal cancer, indicating TNS1 could potentially be a therapeutic target [39]. MiR-31-5p serves as an oncosuppressor miRNA and increases the sensitivity of bladder cancer cells to mitomycin C [40,41]. EGCG also exerted cytotoxic effect to bladder cancer cells with similar efficacy to mitomycin C [10]. This suggests that EGCG can be a potent adjunctive compound to mitomycin C in bladder cancer treatment through regulating miR-31-5p and its potential target TNS1. However, the role of miR-642a-5p in bladder cancer remains unclear, and further investigation is needed to clarify the role of miR-642a-5p-TNS1 interaction in bladder cancer in response to EGCG treatment.

In our current study, DLG2 (discs large homolog 2) potentially regulated by miR-1226-3p and miR-484, along with SCRIB and SMAD1, were associated with Hippo signaling (*p* = 6.74 × 10^−3^). The effect of EGCG in cancer treatment was validated from in vitro and in vivo studies [4]. The Hippo signaling pathway is found dysregulated in bladder cancer, causing cancer progression and chemotherapy resistance [42]. EGCG was proposed to affect cell proliferation and apoptosis through Hippo signaling pathway in oral cancer [43], but has not been studied in bladder cancer. While the role of DLG2 as a tumor suppressor gene in osteosarcoma [44] and miRNA target in ovarian cancer inhibiting cell migration [45] were reported, its involvement in bladder cancer remains unclear.

There are some limitations in the current study. Firstly, the current results were based on results from bladder cancer cell line, BFTC-905. Since genomic variability is an important issue in urothelial carcinoma, future study will be conducted on other bladder cancer cell lines and primary cells from patients with urothelial carcinoma for further investigation. In addition, experimental validation of potential targets and miRNAs in EGCG-treated bladder cancer cells is needed. Moreover, it is important to investigate the changes in gene expression in clinical specimens of patients with different bladder cancer stages to determine the therapeutic effect of EGCG.

## 5. Conclusions

In summary, 108 differentially expressed genes and 22 candidate genes with potential miRNA interactions in EGCG-treated bladder TCC cells were identified. These genes were mainly involved in NAD biogenesis, inflammatory response, and oxidation-reduction metabolism. Moreover, several miRNA–mRNA interactions that were potentially altered with EGCG treatment were identified. These findings may provide insights into novel therapeutic targets and underlying mechanisms of action of EGCG treatment in bladder cancer.

## Figures and Tables

**Table 1:** Candidate genes with potential miRNA interactions of epigallocatechin gallate (EGCG)-treated bladder transitional cell carcinoma (TCC) cells.

Gene Symbol	Gene Name	EGCG FPKM	Control FPKM	Fold-Change (EGCG/Control)
*GTF2H2C*	GTF2H2 family member C	1.38	0.00	137677.00
*PRRT2*	proline rich transmembrane protein 2	21.44	0.17	126.48
*RASA4*	RAS p21 protein activator 4	1.81	0.17	10.92
*TMEM92*	transmembrane protein 92	1.85	0.53	3.52
*RGPD5*	RANBP2-like and GRIP domain containing 5	2.70	0.78	3.47
*SEPT3*	septin 3	0.70	0.20	3.42
*MGAT5B*	mannosyl (alpha-1,6-)-glycoprotein beta-1,6-N-acetyl-glucosaminyltransferase, isozyme B	0.69	0.23	2.95
*GOLGA8B*	golgin A8 family member B	2.59	0.93	2.79
*TSPAN2*	tetraspanin 2	2.23	0.91	2.45
*SBNO2*	strawberry notch homolog 2	2.08	0.91	2.28
*PPM1K*	protein phosphatase, Mg2+/Mn2+ dependent 1K	1.31	2.66	−2.04
*FCGRT*	Fc fragment of IgG receptor and transporter	2.05	4.95	−2.41
*CDON*	cell adhesion associated, oncogene regulated	0.91	2.25	−2.47
*NMNAT2*	nicotinamide nucleotide adenylyltransferase 2	1.06	3.45	−3.27
*DLG2*	discs large MAGUK scaffold protein 2	0.65	2.18	−3.33
*ARRB1*	arrestin beta 1	0.34	1.37	−4.01
*RBPMS*	RNA binding protein with multiple splicing	0.28	1.24	−4.45
*TXNDC2*	thioredoxin domain containing 2	0.13	0.68	−5.06
*TNS1*	tensin 1	0.10	0.72	−6.96
*GIPR*	gastric inhibitory polypeptide receptor	0.51	4.77	−9.44
*NDUFS1*	NADH: ubiquinone oxidoreductase core subunit S1	0.07	1.81	−26.61
*KMO*	kynurenine 3-monooxygenase	0.02	0.61	−33.32

EGCG, epigallocatechin gallate.

**Table 2:** Top five canonical pathways and diseases and disorders of 22 candidate genes.

**Canonical Pathway**	***p*-Value**	**Overlap**	**Molecules**
NAD biosynthesis II	7.73 × 10^−5^	14.3%	*KMO*, *NMNAT2*
NAD biosynthesis III	5.67 × 10^−3^	16.7%	*NMNAT2*
NAD biosynthesis from 2-amino-3-carboxymuconate semialdehyde	6.62 × 10^−3^	14.3%	*KMO*
Thioredoxin pathway	6.62 × 10^−3^	14.3%	*TXNDC2*
NAD salvage pathway III	6.62 × 10^−3^	14.3%	*NMNAT2*
**Diseases and Disorders**	***p*-Value Range**	**Number of Molecules**	
Inflammatory response	3.36 × 10^−2^ to 1.86 × 10^−4^	8	*TSPAN2*, *TNS1*, *MGAT5B*, *SBNO2*, *GIPR*, *FCGRT*, *ARRB1*, *KMO*
Cancer	4.54 × 10^−2^ to 2.86 × 10^−4^	21	
Organismal injury and abnormalities	4.54 × 10^−2^ to 2.86 × 10^−4^	21	
Reproductive system disease	1.49 × 10^−2^ to 6.33 × 10^−4^	15	
Dermatological diseases and conditions	2.62 × 10^−2^ to 8.26 × 10^−4^	14	

**Table 3:** Networks associated with 22 candidate genes in EGCG-treated bladder TCC cells.

	Top Diseases and Functions	Score	Focus Molecules	Molecules in Network
1	Nervous System Development and Function, Tissue Morphology, Neurological Disease	24	10	*APP*, ↓***ARRB1***, Akt, ↓***CDON***, Cytochrome bc1, *ECE2*, ↓***GIPR***, *HNRNPL*, Jnk, *MYC*, *NDUFB8*, ↓***NDUFS1***, *NDUFS4*, *NEDD4*, *NFATC2*, ↓***NMNAT2***, *NTRK1*, Nedd4, ↓***PPM1K***, ↑***RASA4***, ↑***RGPD5***, ↑***TMEM92***, ↑***TSPAN2***, Trhr2, *XPO1*, miR-1229-3p, miR-27a-3p, miR-3691-5p, miR-376b-5p, miR-4435, miR-4752, miR-550b-3p, miR-6811-3p, miR-8056, quinolinic acid
2	Inflammatory Response, Antimicrobial Response, Cellular Development	24	10	*CDH1*, ↓***DLG2***, *EP300*, *F2RL1*, ↓***FCGRT***, *GOLGA8A*/↑***GOLGA8B***, ↑***GTF2H2C***, *GTF2H2C_2*, Holo RNA polymerase II, *IFNG*, *IL6*, ↓***KMO***, *MDM2*, *NFATC2*, ↑***PRRT2***, ↑***SBNO2***, ↑***SEPT3***, *SRC*, ↓***TNS1***, ↓***TXNDC2***, *XPO1*, miR-103b, miR-1243, miR-1281, miR-1307-3p, miR-20a-3p, miR-217-5p, miR-3060-5p, miR-3187-3p, miR-4638-5p, miR-4684-3p, miR-4707-3p, miR-4743-3p, miR-499b-5p, miR-924
3	Connective Tissue Development and Function, Embryonic Development, Organismal Development	4	2	*CAMK2B*, *CREB5*, *DMRT3*, *FNDC11*, *HOXA1*, *KRTAP12-2*, *KRTAP19-5*, ↑***MGAT5B***, *OTX1*, *PITX1*, ↓***RBPMS***, *TRIP13*, miR-1285-3p, miR-3473b, miR-4749-3p, miR-485-5p, miR-6132, miR-6850-3p, miR-744-5p

The genes marked in **bold** were the candidate genes identified in EGCG-treated bladder TCC cells.

**Table 4:** Top five canonical pathways and diseases and disorders of differentially expressed genes.

**Canonical Pathway**	***p*-Value**	**Overlap**	**Molecules**
NAD biosynthesis II	1.77 × 10^−3^	14.3%	***KMO*, *NMNAT2***
Hippo signaling	6.74 × 10^−3^	3.5%	***DLG2***, *SCRIB*, *SMAD1*
Dopamine degradation	8.08 × 10^−3^	6.7%	*ALDH1A1*, *SULT1A3*
Spliceosomal cycle	9.01 × 10^−3^	50.0%	*U2AF1*
Asparagine degradation I	9.01 × 10^−3^	50.0%	*ASPG*
**Diseases and Disorders**	***p*-Value Range**	**Number of Molecules**	
Cancer	4.86 × 10^−2^ to 1.12 × 10^−4^	94	
Organismal injury and abnormalities	4.86 × 10^−2^ to 1.12 × 10^−4^	96	
Infectious diseases	4.42 × 10^−2^ to 1.08 × 10^−3^	3	
Cardiovascular disease	4.42 × 10^−2^ to 1.62 × 10^−3^	13	
Hereditary disorder	4.42 × 10^−2^ to 1.62 × 10^−3^	25	

The genes marked in **bold** were the candidate genes identified in EGCG-treated bladder TCC cells.

**Table 5:** Enrichment analysis of differentially expressed genes in Database for Annotation, Visualization and Integrated Discovery (DAVID) database.

Biological Process	Genes	*p* Value	Fold Enrichment
Oxidation-reduction process	*P3H2, ALDH1A1, PXDN, **TXNDC2**, PYROXD2, NCF1, **KMO**, SDR9C7, PRODH*	0.0108	2.934
Positive regulation of IκB kinase/NFκB signaling	*CD36, EEF1D, GPR89A, S100A12*	0.0497	4.795
NAD metabolic process	***NMNAT2**, **KMO***	0.0549	35.093
xenobiotic metabolic process	*NAT1, SULT1A3, S100A12*	0.0606	7424
carboxylic acid metabolic process	*GAD1, PDXDC1*	0.0646	29.694
response to fatty acid	*CD36, **GIPR***	0.0836	22.707
superoxide metabolic process	*IMMP2L, NCF1*	0.0976	19.301
KEGG pathway	Genes	*p* value	Fold Enrichment
Metabolic pathways	*ALDH1A1, **NMNAT2**, **MGAT5B**, NAT1, **KMO**, PLCD1, ITPK1, GAD1, POLR2J2, **NDUFS1**, PRODH*	0.0651	1.774

The genes marked in **bold** were the candidate genes identified in EGCG-treated bladder TCC cells.

**Table 6:** Validation of potentially altered miRNA–mRNA interactions in EGCG-treated bladder TCC cells.

Putative mRNA	mRNA Fold Change (EGCG/Ctrl)	Predicted miRNA	miRNA Fold Change (EGCG/Ctrl)	miRmap Score	TargetScan	miRDB	Seed Region of 3′UTR
***ARRB1***	−4.01	hsa-miR-185-3p	3.92	98.87	Yes	Yes	815–821, 1723–1729
*ARRB1*	−4.01	hsa-miR-3139	2.88	98.04	Yes	No	
*RBPMS*	−4.45	hsa-miR-3176	2.17	97.23	Yes	No	
***MGAT5B***	2.95	hsa-miR-3116	−4.65	98.69	Yes	Yes	201–207, 492–499, 537–543, 1263–1270
*MGAT5B*	2.95	hsa-miR-6724-5p	−3.96	99.88	Yes	No	
*NDUFS1*	−26.61	hsa-miR-1285-3p	3.43	97.99	Yes	No	
*TNS1*	−6.96	hsa-miR-1285-3p	3.43	98.55	Yes	No	
*TNS1*	−6.96	hsa-miR-18a-3p	3.13	99.36	Yes	No	
***TNS1***	−6.96	hsa-miR-31-5p	2.14	97.84	Yes	Yes	2786–2793, 4195–4202
*TNS1*	−6.96	hsa-miR-3176	2.17	99.13	Yes	No	
***TNS1***	−6.96	hsa-miR-642a-5p	8.27	99.85	Yes	Yes	137–144, 3071–3077, 3157–3164, 3437–3443, 4219–4225
***DLG2***	−3.33	hsa-miR-1226-3p	2.28	99.63	Yes	Yes	3812–3819, 3825–3831, 3846–3853
***DLG2***	−3.33	hsa-miR-484	2.68	80.73	Yes	Yes	1104–1110
*NMNAT2*	−3.27	hsa-miR-185-3p	3.92	98.12	Yes	No	
*NMNAT2*	−3.27	hsa-miR-93-3p	2.64	97.35	Yes	No	
*KMO*	−33.32	hsa-miR-18a-3p	3.13	97.34	Yes	No	
***PPM1K***	−2.04	hsa-miR-22-3p	2.04	97.03	Yes	Yes	299–306

The genes marked in **bold** were the miRNA–mRNA interactions validated in all three miRNA prediction databases.

## References

[1] Babjuk M., Burger M., Comperat E.M., Gontero P., Mostafid A.H., Palou J., van Rhijn B.W.G., Roupret M., Shariat S.F., Sylvester R. (2019). European Association of Urology Guidelines on Non-Muscle-Invasive Bladder Cancer (TaT1 and Carcinoma In Situ)—2019 Update. Eur. Urol..

[2] Tan L.B., Chen K.T., Guo H.R. (2008). Clinical and epidemiological features of patients with genitourinary tract tumour in a blackfoot disease endemic area of Taiwan. BJU Int..

[3] Alfred Witjes J., Lebret T., Comperat E.M., Cowan N.C., De Santis M., Bruins H.M., Hernandez V., Espinos E.L., Dunn J., Rouanne M. (2017). Updated 2016 EAU Guidelines on Muscle-Invasive and Metastatic Bladder Cancer. Eur. Urol..

[4] Colomer R., Sarrats A., Lupu R., Puig T. (2017). Natural Polyphenols and their Synthetic Analogs as Emerging Anticancer Agents. Curr. Drug Targets.

[5] Ozcan A., Korkmaz A., Oter S., Coskun O. (2005). Contribution of flavonoid antioxidants to the preventive effect of mesna in cyclophosphamide-induced cystitis in rats. Arch. Toxicol..

[6] Bazi T., Hajj-Hussein I.A., Awwad J., Shams A., Hijaz M., Jurjus A. (2013). A modulating effect of epigallocatechin gallate (EGCG), a tea catechin, on the bladder of rats exposed to water avoidance stress. Neurourol. Urodyn..

[7] Coyle C.H., Philips B.J., Morrisroe S.N., Chancellor M.B., Yoshimura N. (2008). Antioxidant effects of green tea and its polyphenols on bladder cells. Life Sci..

[8] Feng C., Ho Y., Sun C., Xia G., Ding Q., Gu B. (2017). Epigallocatechin gallate inhibits the growth and promotes the apoptosis of bladder cancer cells. Exp. Ther. Med..

[9] Luo K.W., Lung W.Y., Chun X., Luo X.L., Huang W.R. (2018). EGCG inhibited bladder cancer T24 and 5637 cell proliferation and migration via PI3K/AKT pathway. Oncotarget.

[10] Selman S.H., Keck R.W. (2011). A comparative study of the inhibiting effects of mitomycin C and polyphenolic catechins on tumor cell implantation/growth in a rat bladder tumor model. J. Urol..

[11] Dettlaff K., Stawny M., Ogrodowczyk M., Jelinska A., Bednarski W., Watrobska-Swietlikowska D., Keck R.W., Khan O.A., Mostafa I.H., Jankun J. (2017). Formulation and characterization of EGCG for the treatment of superficial bladder cancer. Int. J. Mol. Med..

[12] Gee J.R., Saltzstein D.R., Kim K., Kolesar J., Huang W., Havighurst T.C., Wollmer B.W., Stublaski J., Downs T., Mukhtar H. (2017). A Phase II Randomized, Double-blind, Presurgical Trial of Polyphenon E in Bladder Cancer Patients to Evaluate Pharmacodynamics and Bladder Tissue Biomarkers. Cancer Prev Res..

[13] Bolger A.M., Lohse M., Usadel B. (2014). Trimmomatic: A flexible trimmer for Illumina sequence data. Bioinformatics.

[14] Kim D., Langmead B., Salzberg S.L. (2015). HISAT: A fast spliced aligner with low memory requirements. Nat. Methods.

[15] Trapnell C., Roberts A., Goff L., Pertea G., Kim D., Kelley D.R., Pimentel H., Salzberg S.L., Rinn J.L., Pachter L. (2012). Differential gene and transcript expression analysis of RNA-seq experiments with TopHat and Cufflinks. Nat. Protoc..

[16] Friedlander M.R., Mackowiak S.D., Li N., Chen W., Rajewsky N. (2012). miRDeep2 accurately identifies known and hundreds of novel microRNA genes in seven animal clades. Nucleic Acids Res..

[17] Kramer A., Green J., Pollard J., Tugendreich S. (2014). Causal analysis approaches in Ingenuity Pathway Analysis. Bioinformatics.

[18] Zhou G., Xia J. (2018). OmicsNet: A web-based tool for creation and visual analysis of biological networks in 3D space. Nucleic Acids Res..

[19] Huang da W., Sherman B.T., Lempicki R.A. (2009). Systematic and integrative analysis of large gene lists using DAVID bioinformatics resources. Nat. Protoc..

[20] Vejnar C.E., Zdobnov E.M. (2012). MiRmap: Comprehensive prediction of microRNA target repression strength. Nucleic Acids Res..

[21] Agarwal V., Bell G.W., Nam J.W., Bartel D.P. (2015). Predicting effective microRNA target sites in mammalian mRNAs. eLife.

[22] Wong N., Wang X. (2015). miRDB: An online resource for microRNA target prediction and functional annotations. Nucleic Acids Res..

[23] Kennedy B.E., Sharif T., Martell E., Dai C., Kim Y., Lee P.W., Gujar S.A. (2016). NAD(+) salvage pathway in cancer metabolism and therapy. Pharmacol. Res..

[24] Nacarelli T., Zhang R. (2019). NAD(+) metabolism controls inflammation during senescence. Mol. Cell Oncol..

[25] Pan L.Z., Ahn D.G., Sharif T., Clements D., Gujar S.A., Lee P.W. (2014). The NAD+ synthesizing enzyme nicotinamide mononucleotide adenylyltransferase 2 (NMNAT-2) is a p53 downstream target. Cell Cycle.

[26] Kusumanchi P., Zhang Y., Jani M.B., Jayaram N.H., Khan R.A., Tang Y., Antony A.C., Jayaram H.N. (2013). Nicotinamide mononucleotide adenylyltransferase2 overexpression enhances colorectal cancer cell-kill by Tiazofurin. Cancer Gene Ther..

[27] Wang W., Hu Y., Yang C., Zhu S., Wang X., Zhang Z., Deng H. (2018). Decreased NAD Activates STAT3 and Integrin Pathways to Drive Epithelial-Mesenchymal Transition. Mol. Cell Proteom. MCP.

[28] Mierzejewska P., Gawlik-Jakubczak T., Jablonska P., Czajkowski M., Kutryb-Zajac B., Smolenski R.T., Matuszewski M., Slominska E.M. (2018). Nicotinamide metabolism alterations in bladder cancer: Preliminary studies. Nucleosides Nucleotides Nucleic Acids.

[29] Zhang Y., Yang D., Zhu J.H., Chen M.B., Shen W.X., He J. (2015). The association between NQO1 Pro187Ser polymorphism and urinary system cancer susceptibility: A meta-analysis of 22 studies. Cancer Invest..

[30] Ma E., Chen P., Wilkins H.M., Wang T., Swerdlow R.H., Chen Q. (2017). Pharmacologic ascorbate induces neuroblastoma cell death by hydrogen peroxide mediated DNA damage and reduction in cancer cell glycolysis. Free Radic Biol. Med..

[31] Kim H.S., Ku J.H. (2016). Systemic Inflammatory Response Based on Neutrophil-to-Lymphocyte Ratio as a Prognostic Marker in Bladder Cancer. Dis Markers.

[32] Sui X., Lei L., Chen L., Xie T., Li X. (2017). Inflammatory microenvironment in the initiation and progression of bladder cancer. Oncotarget.

[33] Xing L., Zhang H., Qi R., Tsao R., Mine Y. (2019). Recent Advances in the Understanding of the Health Benefits and Molecular Mechanisms Associated with Green Tea Polyphenols. J. Agric. Food Chem..

[34] Chen C.Y., Kao C.L., Liu C.M. (2018). The Cancer Prevention, Anti-Inflammatory and Anti-Oxidation of Bioactive Phytochemicals Targeting the TLR4 Signaling Pathway. Int. J. Mol. Sci..

[35] El-Khoury V., Beland M., Schritz A., Kim S.Y., Nazarov P.V., Gaboury L., Sertamo K., Bernardin F., Batutu R., Antunes L. (2018). Identification of beta-arrestin-1 as a diagnostic biomarker in lung cancer. Br. J. Cancer.

[36] Yang Y., Guo Y., Tan S., Ke B., Tao J., Liu H., Jiang J., Chen J., Chen G., Wu B. (2015). β-Arrestin1 enhances hepatocellular carcinogenesis through inflammation-mediated Akt signalling. Nat. Commun..

[37] Kallifatidis G., Smith D.K., Morera D.S., Gao J., Hennig M.J., Hoy J.J., Pearce R.F., Dabke I.R., Li J., Merseburger A.S. (2019). β-Arrestins Regulate Stem Cell-Like Phenotype and Response to Chemotherapy in Bladder Cancer. Mol. Cancer Ther..

[38] Liu C., Li G., Ren S., Su Z., Wang Y., Tian Y., Liu Y., Qiu Y. (2017). miR-185-3p regulates the invasion and metastasis of nasopharyngeal carcinoma by targeting WNT2B in vitro. Oncol. Lett..

[39] Zhou H., Zhang Y., Wu L., Xie W., Li L., Yuan Y., Chen Y., Lin Y., He X. (2018). Elevated transgelin/TNS1 expression is a potential biomarker in human colorectal cancer. Oncotarget.

[40] Bi J., Liu H., Cai Z., Dong W., Jiang N., Yang M., Huang J., Lin T. (2018). Circ-BPTF promotes bladder cancer progression and recurrence through the miR-31-5p/RAB27A axis. Aging.

[41] Xu T., Qin L., Zhu Z., Wang X., Liu Y., Fan Y., Zhong S., Wang X., Zhang X., Xia L. (2016). MicroRNA-31 functions as a tumor suppressor and increases sensitivity to mitomycin-C in urothelial bladder cancer by targeting integrin α5. Oncotarget.

[42] Xia J., Zeng M., Zhu H., Chen X., Weng Z., Li S. (2018). Emerging role of Hippo signalling pathway in bladder cancer. J. Cell Mol. Med..

[43] Li A., Gu K., Wang Q., Chen X., Fu X., Wang Y., Wen Y. (2018). Epigallocatechin-3-gallate affects the proliferation, apoptosis, migration and invasion of tongue squamous cell carcinoma through the hippo-TAZ signaling pathway. Int. J. Mol. Med..

[44] Shao Y.W., Wood G.A., Lu J., Tang Q.L., Liu J., Molyneux S., Chen Y., Fang H., Adissu H., McKee T. (2019). Cross-species genomics identifies DLG2 as a tumor suppressor in osteosarcoma. Oncogene.

[45] Zhuang R.J., Bai X.X., Liu W. (2019). MicroRNA-23a depletion promotes apoptosis of ovarian cancer stem cell and inhibits cell migration by targeting DLG2. Cancer Biol. Ther..

